# PI3Kγ inhibition combined with DNA vaccination unleashes a B-cell-dependent antitumor immunity that hampers pancreatic cancer

**DOI:** 10.1186/s13046-024-03080-1

**Published:** 2024-06-01

**Authors:** Claudia Curcio, Gianluca Mucciolo, Cecilia Roux, Silvia Brugiapaglia, Alessandro Scagliotti, Giorgia Guadagnin, Laura Conti, Dario Longo, Demis Grosso, Mauro Giulio Papotti, Emilio Hirsch, Paola Cappello, Judith A. Varner, Francesco Novelli

**Affiliations:** 1 Department of Molecular Biotechnology and Health Sciences, University of Torino, Piazza Nizza 44Bis, 10126 Turin, Italy; 2 Molecular Biotechnology Center, University of Torino, Turin, Italy; 3 Institute of Biostructures and Bioimaging (IBB), National Research Council of Italy (CNR), Turin, Italy; 4 Pathology Unit, Department of Medical Sciences, University of Torino, AOU Città Della Salute E Della Scienza Di Torino, Turin, Italy; 5 Moores Cancer Center, Department of Pathology, University of California, San Diego, CA USA

## Abstract

Phosphoinositide-3-kinase γ (PI3Kγ) plays a critical role in pancreatic ductal adenocarcinoma (PDA) by driving the recruitment of myeloid-derived suppressor cells (MDSC) into tumor tissues, leading to tumor growth and metastasis. MDSC also impair the efficacy of immunotherapy. In this study we verify the hypothesis that MDSC targeting, via PI3Kγ inhibition, synergizes with α-enolase (ENO1) DNA vaccination in counteracting tumor growth.

Mice that received ENO1 vaccination followed by PI3Kγ inhibition had significantly smaller tumors compared to those treated with ENO1 alone or the control group, and correlated with i) increased circulating anti-ENO1 specific IgG and IFNγ secretion by T cells, ii) increased tumor infiltration of CD8^+^ T cells and M1-like macrophages, as well as up-modulation of T cell activation and M1-like related transcripts, iii) decreased infiltration of Treg FoxP3^+^ T cells, endothelial cells and pericytes, and down-modulation of the stromal compartment and T cell exhaustion gene transcription, iv) reduction of mature and neo-formed vessels, v) increased follicular helper T cell activation and vi) increased “antigen spreading”, as many other tumor-associated antigens were recognized by IgG2c “cytotoxic” antibodies. PDA mouse models genetically devoid of PI3Kγ showed an increased survival and a pattern of transcripts in the tumor area similar to that of pharmacologically-inhibited PI3Kγ-proficient mice. Notably, tumor reduction was abrogated in ENO1 + PI3Kγ inhibition-treated mice in which B cells were depleted.

These data highlight a novel role of PI3Kγ in B cell-dependent immunity, suggesting that PI3Kγ depletion strengthens the anti-tumor response elicited by the ENO1 DNA vaccine.

**Supplementary Information:**

The online version contains supplementary material available at 10.1186/s13046-024-03080-1.

## Statement of significance

The combined treatment with ENO1 DNA vaccine and PI3Kγ inhibition robustly triggers anti-tumor immunity against PDA by increasing the cooperation between T helper follicular and B cells.

## Introduction

Pancreatic ductal adenocarcinoma (PDA) has one of the poorest prognoses among all cancers [1]. Although 10–15% of patients are candidates for surgical resection, recurrence is frequent and the overall 5-year survival rate is only around 10% [2, 3]. Setting up new therapies for PDA is an urgent medical need due to the late-stage at which PDA is diagnosed, its aggressive malignancy and the lack of effective treatments [4, 5].

To find out novel targets for PDA immunotherapy we employed SERological Proteome Analysis to identify Tumor Associated Antigens (TAA) recognized by autoantibodies in PDA patient sera [6]. We identified around a dozen TAA, and we focused our attention on ENO1, a glycolytic enzyme that also acts as a plasminogen receptor that is overexpressed in PDA but not in the normal pancreas [6].

A number of data we obtained prompted us to develop ENO1 DNA as a therapeutic vaccine for PDA and in particular: i) the presence of circulating IgG autoantibodies to ENO1 and its phosphorylated isoforms in the sera in more than 60% of PDA patients but not in normal subjects [6],; ii) the ability of T lymphocytes from PDA patients to secrete IFNγ following stimulation with recombinant ENO1 correlated with the presence of circulating antibodies to ENO1 [7]; iii) the stimulation of T lymphocytes with recombinant ENO1 induced the generation of specific Cytotoxic T Lymphocytes (CTL) that kill pancreatic cancer cells in vitro and in vivo cells but spare normal cells that expressed low levels of ENO1 [7]; iv) the ENO1 targeting by monoclonal antibody or silencing inhibits pancreatic cancer cell migration in vitro and in vivo [8–10]. Based on these results, we have shown that ENO1 DNA vaccination prolonged the survival of genetically engineered mice that spontaneously develop PDA by eliciting an integrated humoral and cellular immune response to ENO1 [11]. Unfortunately, the ENO1 DNA vaccine does not eradicate the tumor; it causes an initial growth inhibition, but the tumor returns to proliferate again, especially when T regulatory (Treg) and myeloid derived suppressor cells (MDSC) are present in the tumor mass [12].

Phosphoinositide-3-kinase γ isoform (PI3Kγ) is expressed in human and murine tumor-associated macrophages and myeloid cells and is responsible for an increase in the suppressive microenvironment and fibrotic reaction to tumors [13, 14]. We have also shown that in PDA mouse models, targeting of PI3Kγ led to improved survival and responsiveness to standard-of-care chemotherapy, by reprogramming tumor-associated macrophages to stimulate CD8^+^ T cell–mediated tumor suppression and inhibiting tumor cell invasion, metastasis and desmoplasia [14]. This evidence has led us to develop strategies for combined treatments designed to both broaden and sustain the anti-tumor immune response elicited by DNA vaccination and PI3Kγ inhibition [13, 14].

In this study, we hypothesize that the integrated anti-tumor humoral and T cell response induced by the ENO1 DNA vaccine is potentiated by the favorable environment elicited by PI3Kγ ablation. This combination robustly reduced PDA growth by enhancing CD8^+^ T lymphocytes and M1-like cell recruitment into the tumor area, and increased the effector IgG2c subclass humoral response and activation of B cells. These data indicate that combining the ENO1 vaccine with the depletion of PI3Kγ could be a potential new therapy to counteract PDA progression.

## Materials and methods

### In vivo experiments

**Fig. 1:**
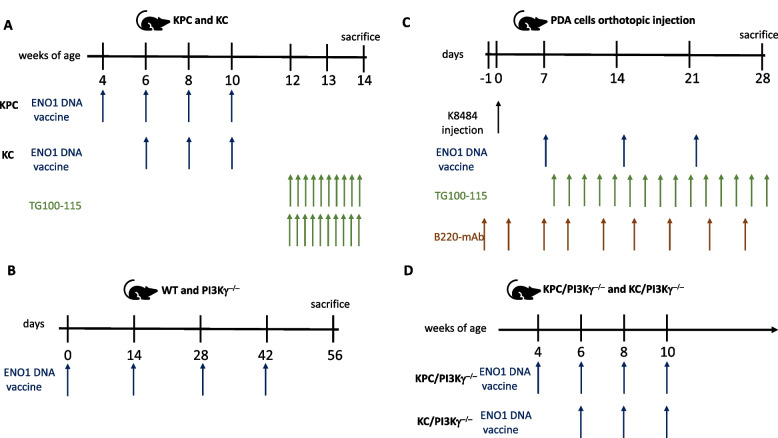
Schematic representation of treatment schedules

Four different mouse models were employed in this study. The first model consisted of mice carrying single-mutated KrasG12D (C57BL/6;129SvJae H-2b, KC) or double-mutated (KrasG12D and Trp53R172H, KPC) (129SvJae H-2b) under the endogenous promoter, and flanked by Lox-STOP-Lox cassettes that were obtained and screened as previously described [11]. KPC or KC mice that spontaneously develop PDA are considered to be a useful tool due to their comparable genomic and physiological tumor biology features between mice and humans [15]. These mice received three or four rounds of ENO1 DNA vaccination until 10 weeks of age and were then treated with the PI3Kγ inhibitor—TG100-115 twice/day for an additional 2 weeks **(**Fig. 1A**)**. TG100-115 is an investigational PI3Kγ inhibitor (Selleckchem, Munich, Germany) and was used at 2.5 mg/kg i.p. twice/day for 2 weeks starting at 12 weeks of age. Mice were monitored by magnetic resonance imaging (Aspect Imaging, Israel) during the treatment period and sacrificed at 14 weeks for tumor lesion evaluation, immune infiltrate, stroma analysis, and cellular and humoral responses.

This model highlights the short-term effect of vaccination followed by PI3Kinhibition. To sacrifice, pancreases were fixed in formalin and embedded in paraffin to analyze tumor lesions by immunohistochemistry or to extract the mRNA (see Supplementary File). Plasmid production and details of vaccination have been previously described [11].

The second model consisted of C57/Bl6 WT and PI3Kγ−/− mice. These mice were vaccinated every 2 weeks for a total of four cycles, then sacrificed at 2 weeks after the final treatment (Fig. 1B). This mouse model was employed to analyze the impact of the absence of PI3Kγ on T helper follicular (Thf) activation and the germinal center (GC) formation elicited by vaccination. Inguinal and popliteal lymph nodes of mice were analyzed by flow cytometry and by Western blotting (see Supplementary file).

The third model was a very aggressive orthotopic PDA mouse model, in which the tumor created a microenvironment similar to that of the primary tumor [16]. Briefly, 1 × 10^5^ cells/mouse of pancreatic tumor cells K8484 (DMEM, 20 mM glutamine, 10% fetal bovine serum- Invitrogen, Milan Italy) at day 0, and were either undepleted or depleted of CD20 cells (Bioxcell, West Lebanon, NH, USA), every 3 days (200 µg/mouse/injected i.p.), starting from the day before tumor injection and continuing until sacrifice (day 30). Mice were treated with ENO1 DNA vaccine and TG100-115 as described in Fig. 1. In these mice the effect of three cycles of vaccination combined with twice/day administration of the PI3Kγ inhibitor—TG100-115 was analyzed. Since in this model the kinetic growth was higher and mouse died as a consequence of the tumor in 1 month from tumor injection, we decided to prolong the treatment with TG100-115, which was well tolerated and no side effects were observed, for a total of 3 weeks. These mice models were employed to assess the effect of B-cell depletion on the immune-response by concomitant administration of the anti-BCD20 monoclonal antibody, ENO1 vaccine and PI3Kγ inhibition (Fig. 1C).

As the PI3Kγ inhibitor—TG100-115 has a short half-life (about 8 h) [17], in order to evaluate the long-term effect of PI3Kγ genetic inhibition on anti-tumor immunity elicited by vaccination, modification of the tumor microenvironment (TME) and survival, a fourth model consisting of genetically PI3Kγ-deleted KC and KPC mice (GEM/ PI3Kγ^−/−^) was employed (Fig. 1D).

Mice were bred and maintained under saprophytic and pathogen-free conditions at the animal facilities of the Molecular Biotechnology Center and treated in accordance with EU and institutional guidelines. This study fully complied with the ethical principles of the Three Rs (3Rs) to Replace, Reduce and Refine the use of animals in research, as required by national and international rules and is provided by Italian Ministry of Health authorization number CC652.119.

### Serological proteome analysis (SERPA)

Sub-confluent K8484 cells were solubilized and 2-DE gels were transferred to nitrocellulose membranes and incubated with sera from mice treated with different therapies. Detailed procedures are described in the Supplementary File.

### Magnetic resonance imaging (MRI)

MRI analysis was performed on mice at 6, 12 and 14 weeks of age. Mice were placed prone in a solenoid Tx/Rx coil with an inner diameter of 35 mm. MR images were acquired on the Aspect M2 MRI scanner (Aspect Imaging, Israel) operating at 1 Tesla. Diffusion-weighted images (DWI) and Apparent Diffusion Coefficient (ADC) analyses were carried out as previously described [18, 19]. Detailed procedures are described in the Supplementary File.

### Dissociation of tissues and FACS analysis

Splenocytes or pancreatic tissue (2 × 10^5^ cells) from differently treated mice were dissociated, washed with PBS/0.2% BSA/0.01% NaN_3_, stained with the following monoclonal antibodies: CD4, CD8, CD107, CD19, CD80, CD45, CD40, PD1, IFNγ stained with the following monoclonal antibodieILγ (all Miltenyi, Germany), CD11b (Thermo Fisher Scientific Waltham, Massachusetts, USA), CD11c, and MHCII (both Biolegend, San Diego, California, USA), and subsequently fixed and permeabilized with Fixation and Permeabilization Solution (eBioscience; Campoverde, Milan, Italy) for 30 min at 4 °C. After washing with a permeabilization buffer, cells were incubated for 30 min with FoxP3 and BCL6 Ab (Biolegend, San Diego, California, USA).

All flow cytometry data were acquired on an Accuri C6 and analyzed using FlowJo software.

### Enzyme-Linked Immunosorbent Spot Assay (ELISpot)

Total splenocytes were stimulated after 4 days in vitro in the presence of 10 μg/mL of rENO1 protein. Recovered T cells were then stimulated with K8484 cells (with a stimulator/effector ratio of 1:100) for 48 h at 37 °C in nitrocellulose plates pre-coated with anti–IFNγ monoclonal antibody. T cells were seeded at 1 × 10^5^ cells/well in triplicate to evaluate specific IFNγ production by ELISpot assay (see Supplementary File).

### Analysis of humoral response

Recombinant human ENO1 (rENO1, Sigma-Aldrich, Milan, Italy) protein was used to evaluate the specific IgG titer in mice by ELISA. Antibody dependent cell cytotoxicity (ADCC) was analyzed using carboxyfluorescein diacetate succinimidyl ester (CFSE; Molecular Probes), as previously reported [20], while serum-binding potential was evaluated using sera from treated mice and K8484 [21]. Details of these experiments are described in the Supplementary File.

### Statistical analysis

The one-way ANOVA followed by multiple comparisons test was used to evaluate statistical differences between treatments. Kaplan–Meier survival curves were created using GraphPad software (Prism 7, La Jolla, CA) and evaluated with the log-rank Mantel–Cox test.

## Results

### ENO1 DNA vaccination followed by pharmacological inhibition of PI3Kγ reduces tumor growth by affecting the immune and stromal compartment

**Fig. 2:**
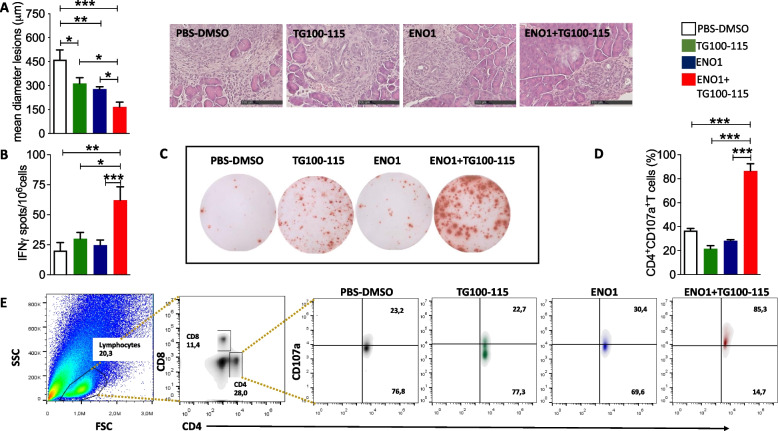
Effect of ENO1 DNA vaccination and PI3Kγ inhibition in KPC mice. Evaluation of the mean tumor lesion diameter in treated mice sacrificed at 14 weeks of age, and representative images of pancreatic tumor lesions (**A**). The scale bar represents 100-μm. ELISpot analysis of IFNγ-secreting cells (indicated as the number of specific spots) from different treatment groups at sacrifice, after in vitro restimulation with recombinant ENO1 (**B**). Representative ELISpot images from different treatments (**C**). Flow cytometry analysis of cytotoxic T cells in pancreatic tumor tissues from mice in different treatment groups (**D**). Representative cytogram analysis (**D**). Representative density plots show: in grey, cells from the PBS-DMSO group, in green, from the TG100-115 inhibitor; in blue, from the ENO1 DNA vaccine; in red, from the ENO1 + TG100-115 combined treatment. The lymphocytes population was identified according to physical parameters (first panel, frame black). For T cell subset analysis, we gated for CD4 and CD8 (second panel), and the expression of CD107a on all CD4.^+^ T cells (third, fourth, fifth and sixth panels) was evaluated. (**E**). Treatment groups: white bars, PBS-DMSO; green bars, TG100-115 inhibitor; blue bars, ENO1 DNA vaccine; red bars, ENO1 + TG100-115, combined treatment. In all experiments, the number of mice per group was between 5 and 10; graphs report the mean ± SEM values, and statistical significance using one way ANOVA test is shown, *p < 0.05, **p < 0.001, and ***p < 0.0001

In KPC mice, both the PI3Kγ inhibitor and vaccine alone reduced the tumor area, with the combined treatment creating an augmented effect (Fig. 2A). Neither treatment affected the number of lesions per mouse or the Ki67 positivity (not shown), suggesting that the combined therapy delayed rather than eradicated PDA progression. Compared to splenocytes from untreated or single therapy-treated mice, combined therapy induced a significant increase in both ENO1-specific IFNγ-secreting (Fig. 2B and C) and cytotoxic CD4^+^CD107a^+^ T cells (Fig. 2D and E), and, to a lesser extent, cytotoxic CD8^+^CD107a^+^ T cells (Supplementary Fig. 1A). To further confirm that the combined treatment expanded ENO1-specific T cells, splenocytes from mice underwent different treatments were re-stimulated with rENO1 (not shown). These ENO1-specific T cells produced high levels of IFNγ similar to those observed after K8484 re-stimulation.

**Fig. 3:**
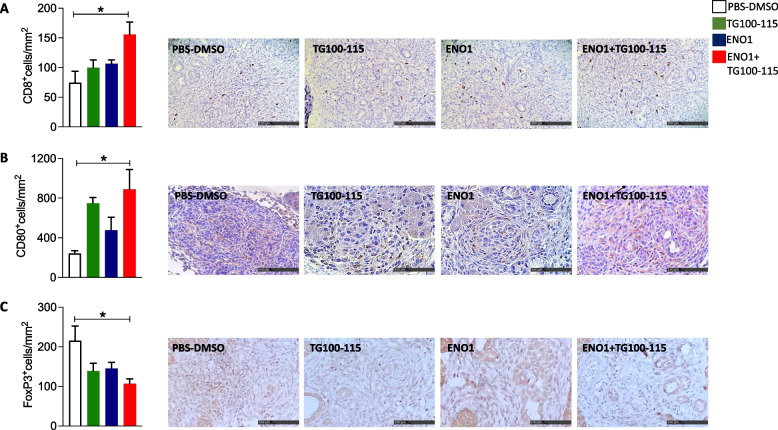
Characterization of immune infiltrates induced by combined treatment. Immunohistochemical staining for CD8 T lymphocytes (**A**), CD80^+^ M1-like macrophages (**B**), and FoxP3.^+^ Treg (**C**) cells in tumor lesions of KPC mice at sacrifice. Representative images from each staining are shown. The scale bar represents 100-μm. Treatment groups: white bars, PBS-DMSO; green bars, TG100-115 inhibitor; blue bars, ENO1 DNA vaccine; red bars, ENO1 + TG100-115, combined treatment. In all experiments, the number of mice per group was between 5 and 10; graphs report the mean ± SEM values, and statistical significance using one way ANOVA test is shown, *p < 0.05, **p < 0.001, and ***p < 0.0001

Tumor infiltrating immune cell analysis by immunohistochemistry showed no changes in the number of CD163^+^ M2-like macrophages (Supplementary Fig. 1B), CD4^+^ (Supplementary Fig. 1C) or B220^+^ (Supplementary Fig. 1D) cells in the different therapy groups. Conversely, the combined therapy caused a statistically significant increase in the number of CD8^+^ T cells (Fig. 3A) and CD80^+^ M1-like macrophages (Fig. 3B) and a reduction of FoxP3^+^ Treg (Fig. 3C) in the tumor area compared to the control group.

**Fig. 4:**
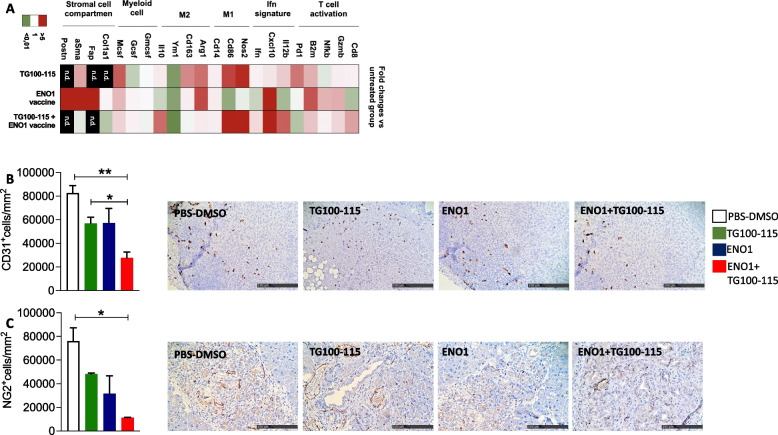
Combined treatment modifies stromal cell compartment and vessel permeability**.** Heat-map represents the fold change of 2^-deltaCt values of the treated groups compared to the untreated group (**A**). mRNA extracts from FFPE tumors of 4–6 mice from each group were analyzed. *Gapdh* was used as a housekeeping gene to obtain delta-Ct values used for the analysis. Green tones represent downregulation of mRNA, red tones upregulation, white tones no change compared to the untreated group. The genes not detected by qPCR are indicated as “n.a.”. Immunohistochemical staining for endothelial CD31^+^ cells (**B**) and neo-formed pericyte NG2.^+^ cells (**C**) in pancreatic tissues. Representative images from each staining are shown. The scale bar represents 100-μm. Treatment groups: white bars, PBS-DMSO; green bars, TG100-115 inhibitor; blue bars, ENO1 DNA vaccine; red bars, ENO1 + TG100-115, combined treatment. In all experiments, the number of mice per group was between 6 and 12; graphs report the mean ± SEM values and statistical significance using one way ANOVA test is shown, *p < 0.05, **p < 0.001, and ***p < 0.0001

Gene transcription analysis of the immune microenvironment by real-time PCR (qPCR) showed that compared to single treatments, the combined therapy induced an increase in *Cd8,* Nuclear factor kB (*Nfkb*) and β2 microglobulin (*Β2m*), and a concomitant downregulation of programmed cell death protein 1 (*Pd1*), suggesting an activated and less exhausted infiltrating T cell phenotype (Fig. 4A), also confirmed by cytofluorimetric analysis showing a significant reduction in CD4^+^ CD25^hi^ FoxP3^+^ PD1^+^ subsets (Supplementary Fig. 2A).

These data fit in well with a concomitant increase in CD4^+^ both IFNγ^+^ and CD107a^+^ T cells (Fig. 2D and E, Supplementary Fig. 1A and Supplementary Fig. 2B and C). An increase in *Ifn* signature, M1-like macrophages and myeloid cell chemo-attractor molecules related transcripts (*Cxcl10* and *Il12b*) was also induced by the combined therapy compared to single treatment (Fig. 4A).

The combined therapy of ENO1 DNA vaccine plus PI3Kγ inhibition significantly reduced the expression of two fibrosis-associated genes, namely *Col1a1* and *aSma***(**Fig. 4A**)**, compared to single treatments. Trichromic staining of pancreatic tissue showed a reduction in collagen induced by PI3Kγ inhibition alone or in combined therapy compared to control and vaccinated-only mice (Supplementary Fig. 1E).

Overall, pharmacological inhibition of PI3Kγ reduced tumor lesions and led to a remodeling of the TME, which was enriched in activated CD8^+^ T cells and classically activated macrophages.

### ENO1 DNA vaccination followed by pharmacological inhibition of PI3Kγ increases tumor immune infiltrate and reduces angiogenesis

Magnetic resonance imaging (MRI) and diffusion-weighted imaging (DWI) were performed on KPC mice treated with single or combined therapy to assess vascular permeability. T2-weighted morphological images detected neoplastic lesions in a small number of mice only (data not shown) and could not be used for treatment monitoring at these early time points. DWI analysis showed no significant differences between control and vaccinated mice at 6 and 11 weeks of age, whereas there was a statistically significant reduction in Apparent Diffusion Coefficient (ADC) in mice receiving combined treatment only at 14 weeks of age (Supplementary Fig. 3A and B). The reduction in the ADC value indicates a reduced diffusion of water molecules in the tissue, which may be caused by a modification of the density and type of cells following immune cell infiltration. Indeed, immunohistochemistry analysis showed an increased immune infiltrate at the tumor site in mice receiving combined treatment (Fig. 3A and B). Immunohistochemistry analysis revealed a decrease in neo-angiogenesis, as shown by the reduction of both endothelial CD31^+^ (Fig. 4B) and neo-formed pericyte NG2^+^ (Fig. 4C) cells in mice receiving combined therapy compared to control mice.

### ENO1 DNA vaccination followed by pharmacological PI3Kγ inhibition increases antigen spreading, IgG2c subclass switch and antibody-dependent cytotoxicity in PDA

**Fig. 5:**
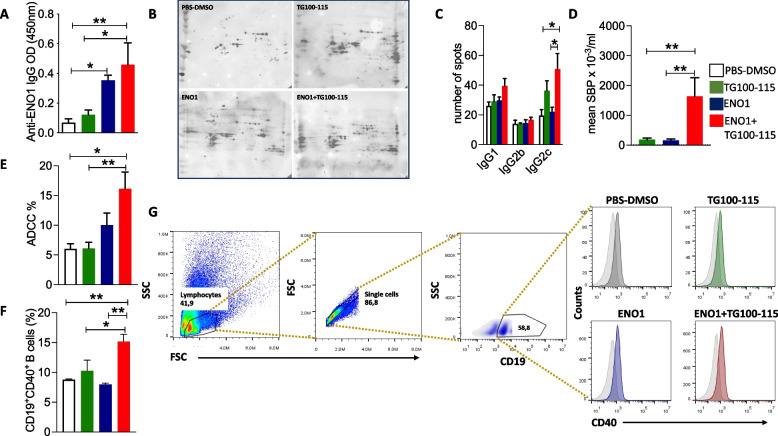
Combined therapy increases the humoral response and induces the antigen spreading effect. ELISA detection of anti-ENO1 IgG titer (referred to as OD) in sera of mice (**A**). IgG2c recognition of the proteome 2-DE map of the K8484 cell line by representative serum from mice treated with different therapies (**B**). IgG1, IgG2b and IgG2c quantitative evaluation of all recognized spots from treated mice (**C**). Serum binding potential (SBP) evaluated by flow cytometry in mice from different treatments collected at 14 weeks of age (**D**). Antibody dependent cellular cytotoxicity (ADCC) percentage evaluated by flow cytometry (**E**). Cytofluorimetric analysis of activated CD19^+^ CD40^+^ B cells in spleen from mice treated with different therapies (**F**). Representative gating strategy to analyze CD40 on CD19 positive cells (**G**). The lymphocyte population was isolated according to physical parameters (first panel), considering only single cells (second panel). According to CD19 positivity we gated B cells (last upper panel), on which we analyzed CD40 positive cells (lower panels). One representative histogram for each group is shown: grey peak, PBS-DMSO; green peak, TG100-115 inhibitor; blue peak, ENO1 DNA vaccine; red peak, ENO1 + TG100-115, combined treatment, light grey peak represents the isotype control. Treatment groups: white bars, PBS-DMSO; green bars, TG100-115 inhibitor; blue bars, ENO1 DNA vaccine; red bars, ENO1+TG100-115 combined treatment. In all experiments, the number of mice per group was between 3 and 10; graphs report the mean±SEM values, and statistical significance using one way ANOVA test is shown, *p < 0.05, **p < 0.001, and ***p < 0.0001

KPC mice vaccinated with ENO1 showed an increased specific anti-ENO1 IgG response compared to controls and mice treated with TG100-115 alone, and to a greater extent with combined therapy (Fig. 5A). Notably, serological analysis of the proteome from the K8484 PDA syngeneic cell line revealed that, compared to single treatment, sera from combined therapy only showed a statistically significant increase in the number of Tumor Associated Antigens (TAA) recognized by IgG2c, the most efficient cytotoxicity inducer of the IgG subclass [22], indicating clear antigen-spreading (AS) [23] (Fig. 5B). Conversely, no differences were observed in the number of TAA recognized by IgG1 and IgG2b subclasses in sera from any of the treatment groups (Fig. 5C). Furthermore, sera from the combined treatment only bound to the cell surface of murine PDA cells (Fig. 5D) and triggered their killing by antibody-dependent cytotoxicity (ADCC) (Fig. 5E), which has been proposed as an effector mechanism of antitumor immunity. In addition, these abilities correlated with a higher percentage of CD19^+^CD40^+^ activated B cells in the spleen (Fig. 5F and G).

### ENO1 DNA vaccination increases T and B cell cooperation and antigen-presenting cell activation and functions in mice genetically devoid of PI3Kγ

**Fig. 6:**
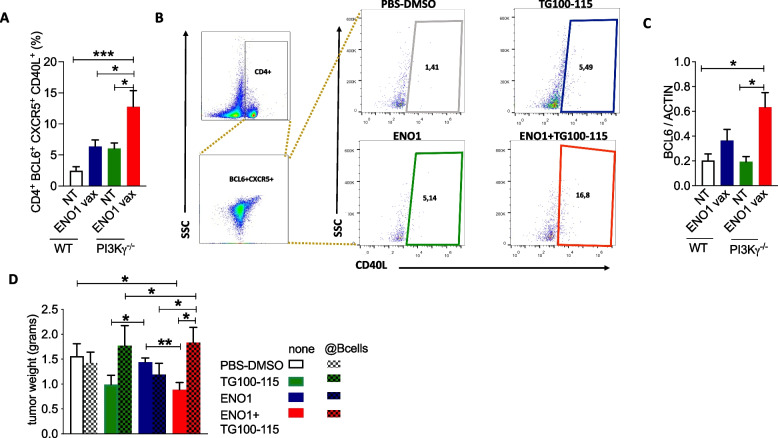
Combined therapy increases activated Thf cells and favors a B cell-dependent antitumor immune response. Flow cytometry analysis (**A**)and representative cytogram (**B**)of activated Thf cells in lymph nodes of C57/Bl6 WT and PI3K γ^-/-^ vaccinated or unvaccinated mice. After the gating of T lymphocytes, we considered the BCL6^+^CXCR5^+^ on CD4^+^ T cells. On the selected Thf we analyzed the percentage of CD40L^+^ cells. Western blot analysis of BCL6 expression in lymph nodes of WT and PI3Kg vaccinated or unvaccinated mice (**C**). Tumor weight of mice that were treated and B cell depleted (checkered bars) or undepleted (**D**). Treatment groups: white bars, PBS-DMSO; green bars, TG100-115 inhibitor; blue bars, ENO1 DNA vaccine; red bars, ENO1+TG100-115 combined treatment. In all experiments, the number of mice per group was between 5 and 12; graphs report the mean±SEM values, and statistical significance using one way ANOVA test is shown, *p < 0.05, **p < 0.001, and ***p < 0.0001

A second model consisting of WT and PI3Kγ^−/−^ mice was employed to assess the effect of vaccination in the absence of PI3Kγ (Fig. 1B) on Thf, B, dendritic (DC) cells and macrophage activation and function. The increased AS and switch to IgG2c induced by the combined therapy showed an increased interplay between T and B cells. To analyze the effect of the absence of PI3Kγ in this crosstalk, the number and activation of CD4^+^BCL6^+^CXCR5^+^ Thf cells in the lymph nodes of untreated or vaccinated WT and PI3Kγ^−/−^ mice were measured by flow cytometry. No differences in Thf percentages were found between untreated or vaccinated WT and PI3Kγ^−/−^ mice (Supplementary Fig. 3C). By contrast, there was an increase in the percentage of activated CD40L^+^ Thf in untreated PI3Kγ^−/−^ compared to WT mice (Fig. 6A and B). In addition, a statistically significant increase in activated CD40L^+^ Thf cells was observed in vaccinated WT compared to untreated mice and, to a greater extent, in vaccinated PI3Kγ^*−/−*^mice (Fig. 6A). As BCL6 is the transcription factor essential for Thf cell differentiation and development, favoring the formation of the GC [24], its expression was analyzed in lymph nodes from vaccinated or unvaccinated WT or PI3Kγ^−/−^ mice. There was an increase in BCL6 expression in vaccinated PI3Kγ^*−/−*^ mice compared to untreated mice (Fig. 6C and Supplementary Fig. 3D), suggesting an increased GC activation.

Cytofluorometric analysis of the splenocytes showed that neither activation marker CD80 nor MHC II were increased in DC (Supplementary Fig. 4A and not shown), macrophages (Supplementary Fig. 4B and C) or B cells from vaccinated or unvaccinated WT mice (not shown). Conversely, in vaccinated PI3Kγ^−/−^mice CD80 alone increased in DC (Supplementary Fig. 4A), together with MHC II in macrophages (Supplementary Fig. 4C), whereas neither CD80 nor MHC II increased in B cells (not shown) compared to WT mice. However, the analysis of WT and PI3Kγ^−/−^ vaccinated mice revealed that in lymph nodes, both macrophages (Supplementary Fig. 4D) and B (Supplementary Fig. 4E) cells from PI3Kγ^−/−^ mice increased in MHC II expression compared to WT, whereas no differences were observed in DC (not shown). This data suggests that the absence of PI3Kγ increases the ability of antigen-presenting cells to present antigens in the spleen and lymph nodes.

### In the orthotopic PDA mouse model, ENO1 DNA vaccination followed by pharmacological PI3Kγ inhibition triggers B-cell dependent anti-tumor immunity

A third orthotopic PDA mouse model (Fig. 1C) was employed to characterize the role of antibodies and B cell-mediated antitumor immunity to counteract PDA progression in mice treated with the combined therapy. When syngeneic K8484 PDA cancer cells were orthotopically injected into the pancreas, it was observed that the combined therapy significantly reduced tumor growth (Fig. 6D) compared to single treatments. Notably, B-cell depletion by anti-CD20 antibody fully reverted the antitumor effect elicited by the combined therapy, confirming the key role of the antibody response (Fig. 6D).

### ENO1 DNA vaccine increased anti-tumor immunity and survival in KC and KPC mice genetically deficient of PI3Kγ

**Fig. 7:**
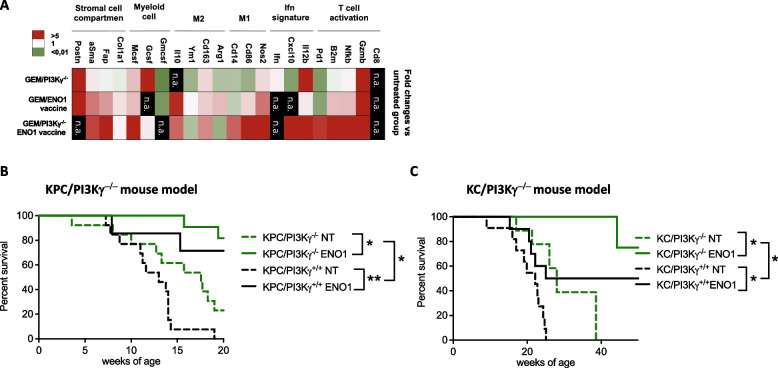
ENO1 DNA vaccination in PI3Kγ^−/−^ deficient mice increases survival, modulates immune and stromal cell compartment. Heat-map represents the fold change of 2^-deltaCt values of the treated groups compared to the untreated group (**A**). mRNA extracts from FFPE tumors of 4–6 mice from each group were analyzed. *Gapdh* was used as a housekeeping gene to obtain delta-Ct values used for the analysis. Green tones represent downregulation of mRNA, red tones upregulation, white tones no change compared to the untreated group. The genes not detected by qPCR are indicated as “n.a.”. Survival curves of KPC or KPC**/**PI3Kγ^−/−^(**B**) and KC or KC/PI3K γ.^−/−^(**C**) untreated (green or black dotted line) or ENO1-vaccinated (green or black line). In all experiments, the number of mice per group was between 7 and 13. Statistical differences using Long-Rank test are shown in the graph, *p < 0.05, **p < 0.001, and ***p < 0.0001

A fourth PDA mice model consisting of KC or KPC mice genetically deleted for PI3Kγ (GEM/PI3Kγ^−/−^) was employed to evaluate the long-term effect of vaccination in the absence of PI3Kγ (Fig. 1D). We vaccinated mice with the same schedule used in proficient KPC mice or, in the case of KC, which display a slower PDA development [11], the vaccination was started 2 weeks later than KPC (Fig. 1A and D). Transcription analysis of the same panel of genes evaluated in pharmacologically PI3Kγ inhibited KPC mice (Fig. 4A) was also performed in GEM/PI3Kγ^−/−^ mice (Fig. 7A). As confirmed by the heatmap showing fold-changes compared to untreated PI3Kγ proficient GEM, vaccination in the absence of PI3Kγ led to a strong up-modulation of T cell activation (*Gzmb, Nnfkb, B2m, Pd1*), IFN signature *(Il12b* and* Cxcl10)* and M1-like macrophage-related (*Nos2, Cd86, Cd14*) genes (Fig. 7A). A marked down-modulation of M2-like macrophage transcripts (*Arg1* and *Ym1*) was also observed, whereas *Cd163* was upregulated in all conditions (Fig. 7A). Conversely, transcripts related to myeloid cell recruitment (*Mcs* and *Gcsf*) and the stromal compartment (*Fap* and *aSma*) were up-modulated by vaccination, which was greatly elevated in the absence of PI3Kγ (Fig. 7A). Indeed, a significant increase in survival was observed in vaccinated PI3Kγ proficient mice, which was greater in PI3Kγ deficient GEM mice. In both KPC and KC mice this difference was limited to the first 15 and 45 weeks, respectively (Fig. 7B and C).

## Discussion

PDA is an immunogenically ‘‘cold’’ tumor because it lacks infiltration of natural T cells, and immune checkpoint blockade failed to increase survival of PDA patients [25]. To convert PDA into a responsive tumor, effective immunotherapy strategies are required to i) increase antigenicity, ii) enhance effector T cell function, and iii) overcome T cell exhaustion and immunosuppressive signals in the TME [26, 27].

The ENO1 DNA vaccine elicits an antitumor response that prolongs survival of spontaneous autochthonous PDA mouse models [11]. However, a few months after the final vaccination, the Treg and MDSC increase again causing uncontrolled growth of the tumor [11], suggesting the need to integrate the vaccine with new strategies aimed at targeting these cells. The effects of the ENO1 DNA vaccine were more pronounced when combined with chemotherapy, inducing AS, as demonstrated by the increase of both IgG production and IFN-γ secretion specific to ENO1 and other TAAs, such as glyceraldheyde-3-phosphate dehydrogenase [28].

PI3Kγ inhibition in PDA-bearing mice reprograms tumor-associated macrophages to stimulate CD8^+^ T cell–mediated tumor suppression and to inhibit tumor cell invasion, metastasis, and desmoplasia [14]. In addition, in orthotopic and KPC mouse PDA models, PI3Kγ inhibition slowed tumor growth and enhanced survival and responsiveness to standard-of-care chemotherapy by altering macrophage transcriptional profiles and thereby activating T cells, indicating that PI3Kγ targeting could provide long-term control of PDA [14]. The drug used in this study to inhibit PI3Kγ was TG100-115, which also has a partial inhibition on the delta isoform of PI3K and has shown remarkable therapeutic efficacy in a broad range of cancers, including non-hematological solid tumors [29]. We cannot rule out that the inhibition of the delta isoform can be induced by TG100-115 treatment. However, the data obtained in PI3Kγ genetically-deficient mice showed superimposable effects on tumor microenvironment modification and anti-tumor effector immunity observed in TG100-115 treated mice, suggesting that the anti-tumor effects of ENO1 vaccination are mainly enhanced by PI3Kγ inhibition.

We have demonstrated that PI3Kγ inhibition combined with the ENO1 DNA vaccine reduces tumor growth, increases immune infiltration at tumor sites, modulates the TME and strongly potentiates the ENO1-specific humoral and cellular responses. The combined treatment increased the percentage of activated B cells and Thf cells leading to antibody switches able to induce an efficient ADCC, strengthened the ability to bind PDA cells and enhanced recognition of TAAs compared to single treatments. Of note, B cell depletion abrogated the anti-tumoral response induced by the combined treatment.

The presence of B cells in tumors predicts an increased patient survival [30], but there are reports of both anti- and pro-tumor roles for B cells [31]. In addition, the presence of tertiary lymphoid structures (TLS) is associated with a favorable response to immunotherapy [32]. The formation of TLS requires long-lasting exposure to inflammatory signals mediated by chemokines (CXCL13, CCL19 and CCL21) and cytokines (IL-1, IL-17, IL-22 and IL-23), along with disease progression [33]. In some observations, it took at least 20 weeks to detect the presence of TLS, and their appearance was random and uncontrolled in mice without any treatment [34, 35]. In KPC mice, which were vaccinated at 4 weeks of age and sacrificed at 14 weeks, there was only a scattered B cell tumor infiltration without any TLS. However, detection of TLS probably requires vaccination of KPC mice bearing larger tumors, as we have previously observed TLS in KC mice vaccinated at 32 weeks of age and analyzed at 40 weeks of age [36].

Successful PDA immunotherapy requires optimization of activated effector T cells [37]. Combined treatment elicited a higher infiltration of immune cells at the tumor site. In particular, qPCR analysis of gene expression showed an increase of CD8^+^ T cells and M1-like macrophages transcripts (Fig. 4A and 7A), that was confirmed by immunohistochemistry staining (Fig. 3A and B). Concomitantly immunohistochemistry revealed a reduction in Treg cells, confirming previous data [14]. Mice treated with the combined therapy showed a significant decrease in the ADC, as demonstrated by MRI analysis (Supplementary Fig. 3A and B), which suggest reduced water diffusion that may be a consequence of the increase in immune cells and macrophage infiltration [38–40]. Finally, qPCR showed that combined treatment induced an increase of gene expression of *ifn* signature markers (Fig. 4A and 7A), which are typically associated with favorable cancer prognosis and immunotherapy response [41–44].

Despite the pro-tumorigenic role of IL10 in PDA [45], it may play a role in the reduction of tumor vascularity in ovarian cancer [46]. We have shown that inhibition of PI3Kγ combined with ENO1 DNA vaccination led to an increased gene transcription of IL10 in PDA mRNA (Fig. 4A and 7A), suggesting that the reduced vascularity showed by immunohistochemistry (Fig. 4B and C) is related to this increase. Since IL10 favors the expansion of Treg cells, which were significantly decreased in mice treated with combined therapy, the precise role of IL10 in inhibiting angiogenesis requires further studies.

Long-term surviving PDA patients can be distinguished by a large inactivated stromal profile, suggesting that stromal modulation may extend survival in PDA [47]. However, therapeutic manipulation of the stroma in PDA has shown mixed results in experimental models [48–50]. Here, there is an increase of stromal cell compartment genes—evaluated by qPCR—in GEM/PI3Kγ ENO1-vaccinated mice (Fig. 7A), suggesting that PI3Kγ is also involved in stromal modulation [13]. Therefore, inhibition of PI3Kγ concomitantly modifies the profibrotic stromal profile and favors higher effector T cell infiltration at the tumor site. However, vaccination in mice genetically devoid of PI3kγ limited the increase of survival to just a few weeks, likely due to still unknown compensatory mechanisms which, in a clinical perspective, are not representative of PDA patients.

The AS, as well as the isotypic switching to IgG2c, the most cytotoxic IgG isotype [22], which promotes the trigger of ADCC, are features of the combined therapy, highlighting the critical role of B cells in regulating the anti-tumor response. Here, the combined therapy increases BCL6 expression and activation of Thf cells in lymph nodes. This creates the conditions for an efficient cooperation with GC B cells to increase the switch to IgG2c, leading to cytotoxicity, favoring the uptake of dying tumor cells by DC, and their cross-presentation of TAA to T cells [51] which, together with the increased activation of B cells, may account for the observed increased AS. In addition, we observed an increase in MHC II expression on macrophages and B cells in lymph nodes and in the spleen, which also fits well with the increased AS induced by combined therapy.

The combined therapy is also effective in PDA orthotopically injected mice that were vaccinated 10 days after the challenge. This represents a model in which the treatment is started when tumors are much more advanced compared to GEM mice, where the treatment started in the presence of PanIn only. Again, in the orthotopic PDA mouse model we demonstrated the central role in the control of the anti-tumor immunity of B cells, as their depletion completely reversed tumor progression triggered by the combined therapy. These data suggest that the in vivo inhibition of PI3Kγ may be an efficacious approach to potentiate the effectiveness of the DNA vaccine in PDA. Notably, some PI3Kγ inhibitors have already been approved in clinical trials, such as Eganelisib (IPI-549), a first-in-class, oral, PI3Kγ inhibitor for tumor patients treated with nivolumab [52, 53] or in combination with atezolizumab, and nab-paclitaxel [54].

In conclusion, our study on preclinical mouse models indicates that targeting of macrophage signaling pathways combined with TAA vaccination may provide novel approaches to improve the long-term survival of pancreatic cancer patients.

### Supplementary Information

Additional file 1. Flow cytometry analysis of cytotoxic CD8a T cells in pancreatic tumor tissues from differently treated mice (A). Immunohistochemical staining of CD163 (B), CD4 (C), B220 (D) in tumor lesions of KPC mice at sacrifice. Collagen deposition evaluated by trichomic staining of pancreatic tissue from treated mice (E). Treatment groups: white bars, PBS-DMSO; green bars, TG100-115 inhibitor; blue bars, ENO1 DNA vaccine; red bars, ENO1+TG100-115 (combined treatment). In all experiments, the number of mice per group was between 5 and 10; graphs report the mean±SEM values and statistical significance using one way ANOVA test is shown.

Additional file 2. Flow cytometry analysis of exhausted (A) and cytotoxic (B, C) CD4 T cells in pancreatic tumor tissue from treated mice. Treatment groups: white bars, PBS-DMSO; green bars, TG100-115 inhibitor; blue bars, ENO1 DNA vaccine; red bars, ENO1+TG100-115 combined treatment. In all experiments, the number of mice per group was between 5 and 10; graphs report the mean±SEM values, and statistical significance using one way ANOVA test is shown, *p < 0.05, **p <0.001, and ***p <0.0001.

Additional file 3. ADC mean (A) and mean perfusion fraction MRI analysis (B) of KPC mice treated with different therapies. Percentage of CD4^+^ BCL6^+^ CXCR5^+^ (Thf) cell in untreated or vaccinated WT and PI3Kγ^−/− ^mice (C). Western blot representative image of BCL6 levels in lymph nodes from vaccinated or unvaccinated WT and PI3Kγ^−/−^ mice (D). Treatment groups: white bars, PBS-DMSO; green bars, TG100-115 inhibitor; blue bars, ENO1 DNA vaccine; red bars, ENO1+TG100-115 (combined treatment). In all experiments, the number of mice per group was between 3 and 10; graphs report the mean±SEM values, and statistical significance using one way ANOVA test is shown, *p < 0.05, **p < 0.001, and *p* <0.0001

Additional file 4. Flow cytometry analysis of activated dendritic cells (A) and macrophages (B**) **and activated macrophages expressing MHC II **(C) **in splenocytes from C57/Bl6 WT and PI3Kg^-/-^ vaccinated or unvaccinated mice. Flow cytometry analysis of MHC II expression of macrophages (D) and B cells (E) in lymph nodes from C57/Bl6 WT and PI3Kg^-/-^ vaccinated or unvaccinated mice. Treatment groups: white bars, PBS-DMSO; green bars, TG100-115 inhibitor; blue bars, ENO1 DNA vaccine; red bars, ENO1+TG100-115 (combined treatment). In all experiments, the number of mice per group was between 3 and 4; graphs report the mean±SEM values, and statistical significance using one way ANOVA test is shown, * *p* < 0.05, ** *p* <0.001, and *** *p* < 0.0001

Additional file 5.

## Data Availability

The data generated in this study are available upon reasonable request from the corresponding authors.
