# Supplementary figures and images for: PI3Kγ inhibition combined with DNA vaccination unleashes a B-cell-dependent antitumor immunity that hampers pancreatic cancer
Source: J Exp Clin Cancer Res. 2024 Jun 1;43:157. doi: 10.1186/s13046-024-03080-1 (PMC11143614; doi:10.1186/s13046-024-03080-1)

**A**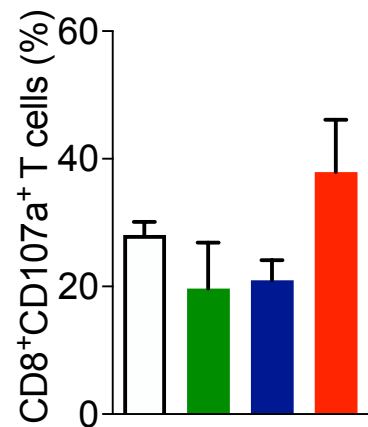**B**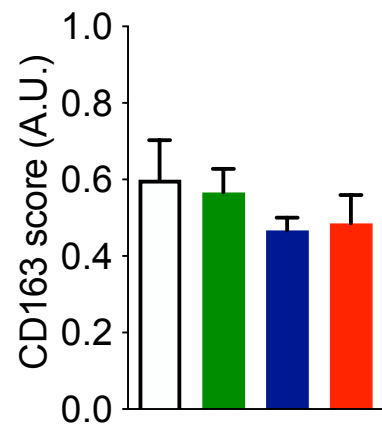**C**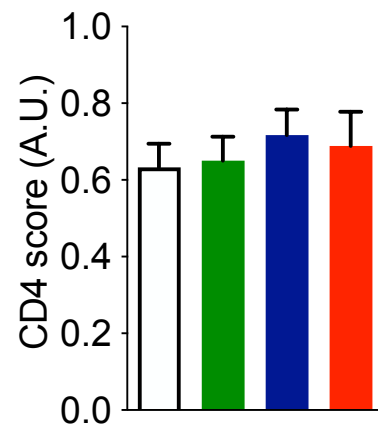

Legend:

- PBS-DMSO
- TG100-115
- ENO1
- ENO1+ TG100-115

**D**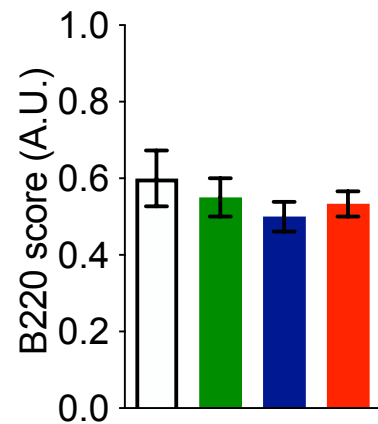**E**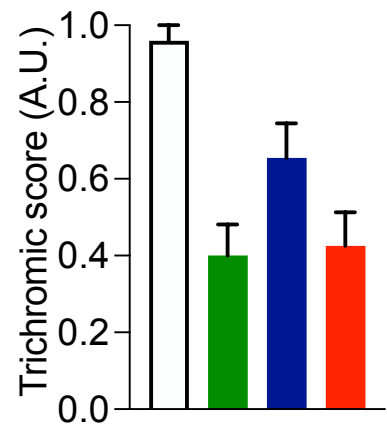

Supplement: Supplementary file 1 — Additional file 1. Flow cytometry analysis of cytotoxic CD8a T cells in pancreatic tumor tissues from differently treated mice (A). Immunohistochemical staining of CD163 (B), CD4 (C), B220 (D) in tumor lesions of KPC mice at sacrifice. Collagen deposition evaluated by trichomic staining of pancreatic tissue from treated mice (E). Treatment groups: white bars, PBS-DMSO; green bars, TG100-115 inhibitor; blue bars, ENO1 DNA vaccine; red bars, ENO1+TG100-115 (combined treatment). In all experiments, the number of mice per group was between 5 and 10; graphs report the mean±SEM values and statistical significance using one way ANOVA test is shown. [file 13046_2024_3080_MOESM1_ESM.pdf]

**A**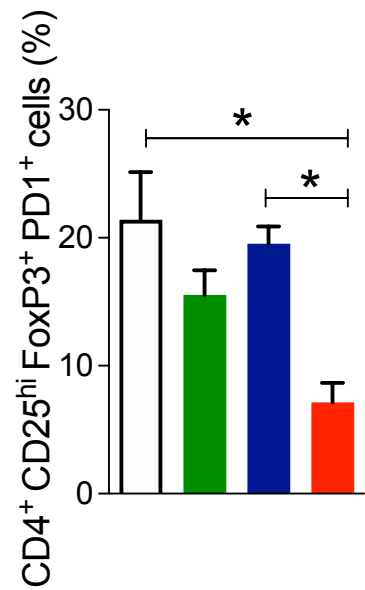**B**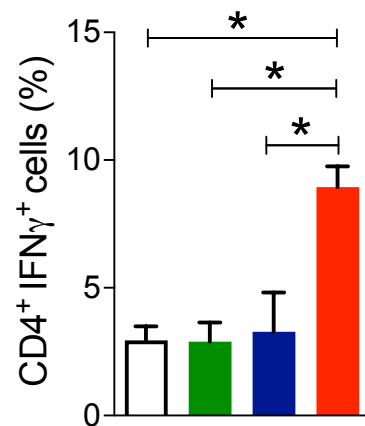**C**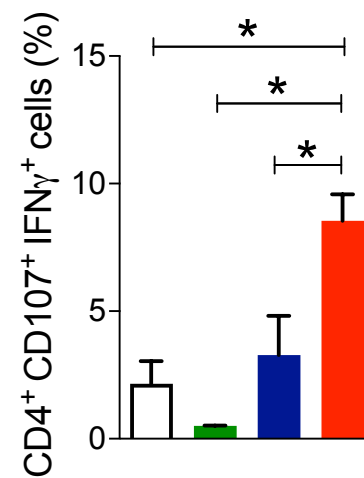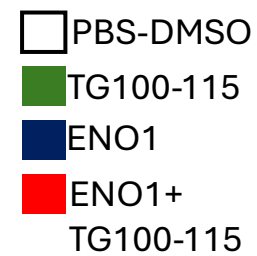

Supplement: Supplementary file 2 — Additional file 2. Flow cytometry analysis of exhausted (A) and cytotoxic (B, C) CD4 T cells in pancreatic tumor tissue from treated mice. Treatment groups: white bars, PBS-DMSO; green bars, TG100-115 inhibitor; blue bars, ENO1 DNA vaccine; red bars, ENO1+TG100-115 combined treatment. In all experiments, the number of mice per group was between 5 and 10; graphs report the mean±SEM values, and statistical significance using one way ANOVA test is shown, *p < 0.05, **p <0.001, and ***p <0.0001. [file 13046_2024_3080_MOESM2_ESM.pdf]

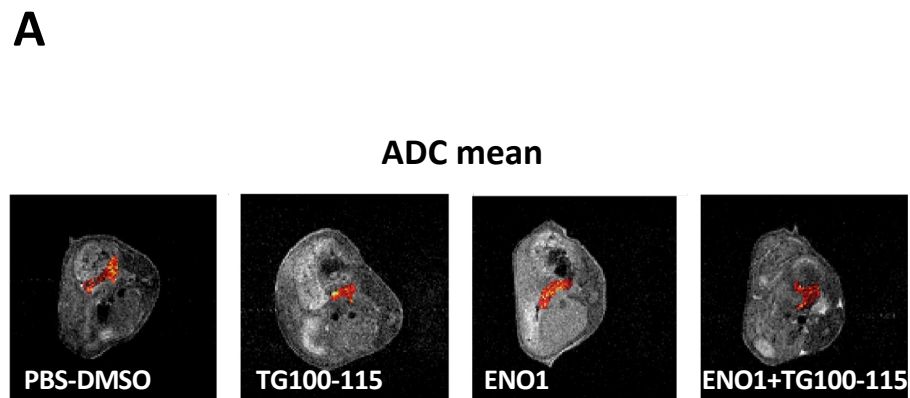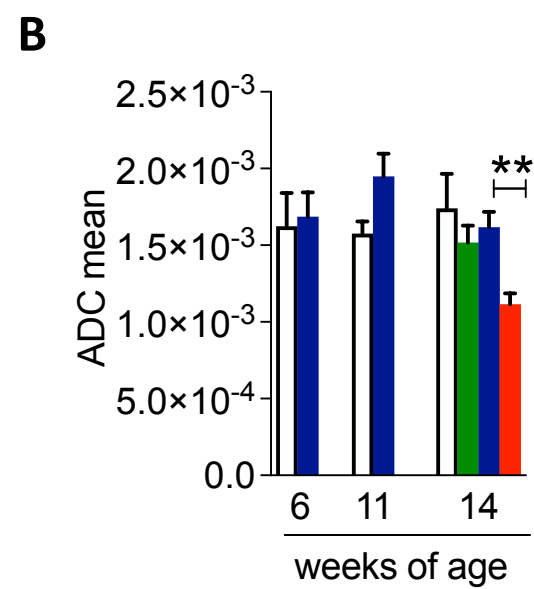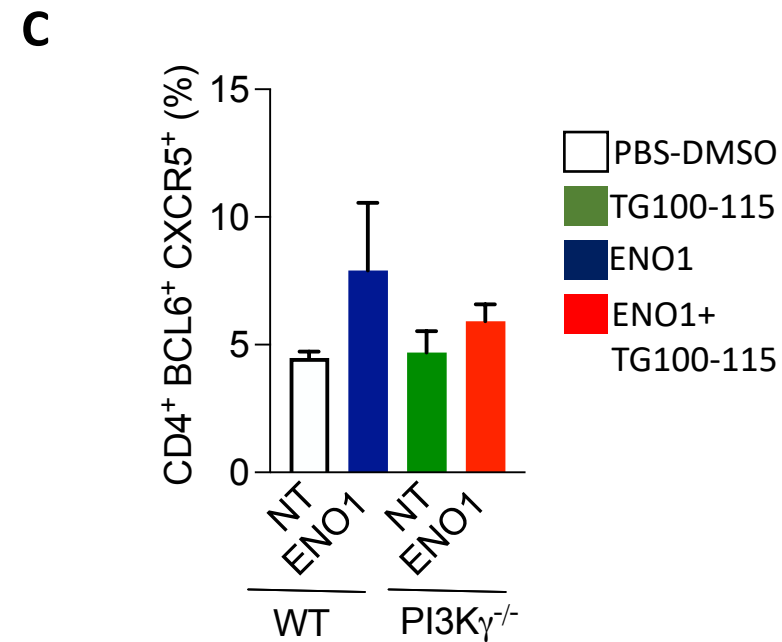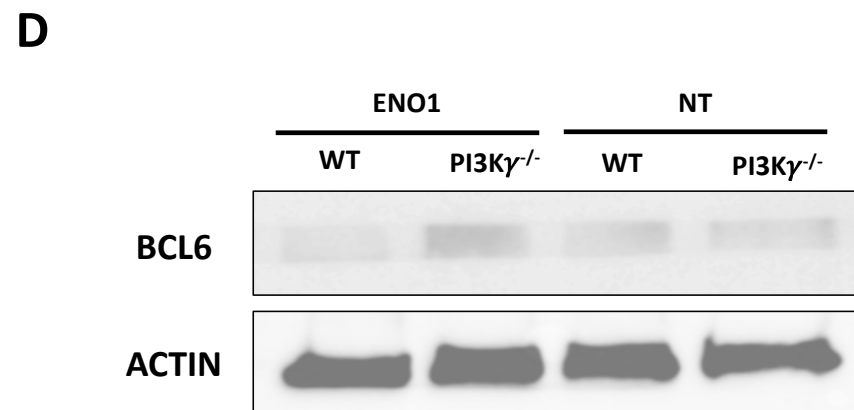

Supplement: Supplementary file 3 — Additional file 3. ADC mean (A) and mean perfusion fraction MRI analysis (B) of KPC mice treated with different therapies. Percentage of CD4+ BCL6+ CXCR5+ (Thf) cell in untreated or vaccinated WT and PI3Kγ−/− mice (C). Western blot representative image of BCL6 levels in lymph nodes from vaccinated or unvaccinated WT and PI3Kγ−/− mice (D). Treatment groups: white bars, PBS-DMSO; green bars, TG100-115 inhibitor; blue bars, ENO1 DNA vaccine; red bars, ENO1+TG100-115 (combined treatment). In all experiments, the number of mice per group was between 3 and 10; graphs report the mean±SEM values, and statistical significance using one way ANOVA test is shown, *p < 0.05, **p < 0.001, and p <0.0001 [file 13046_2024_3080_MOESM3_ESM.pdf]

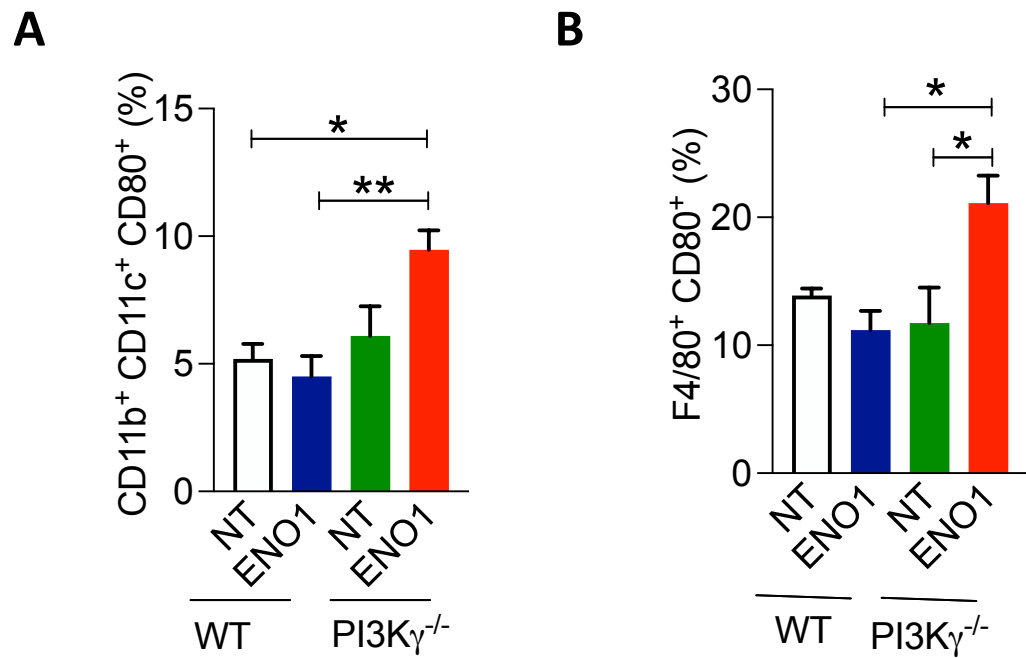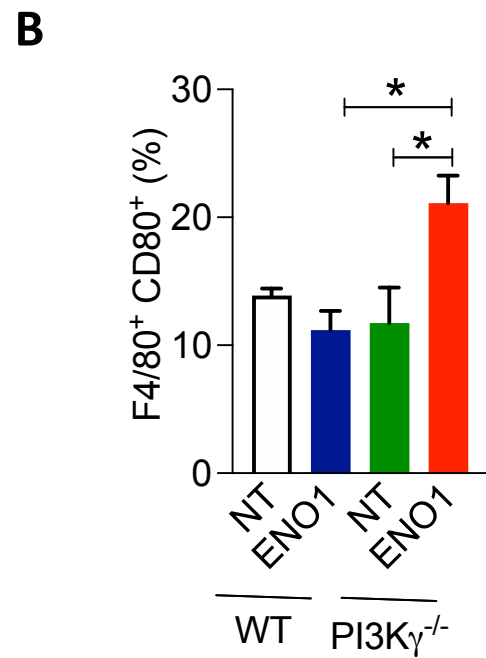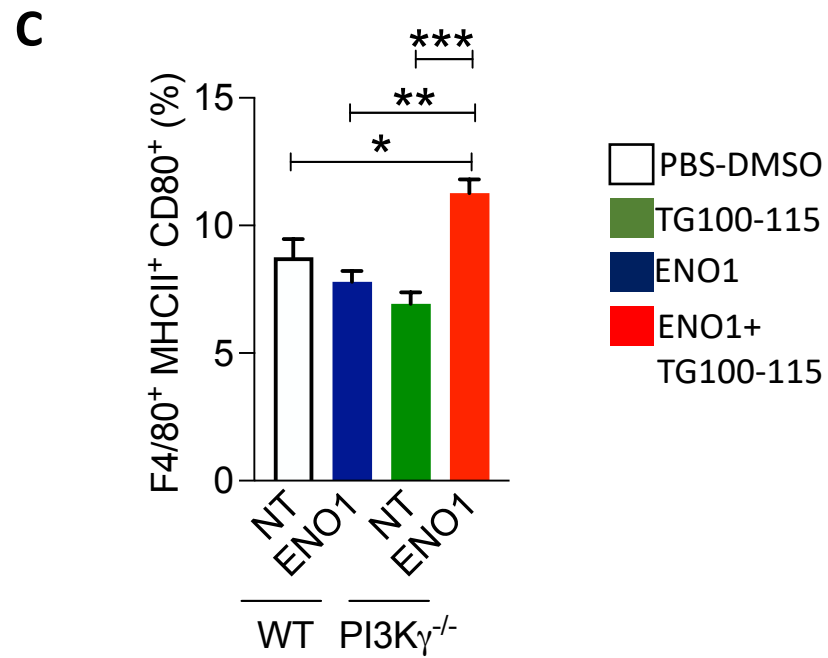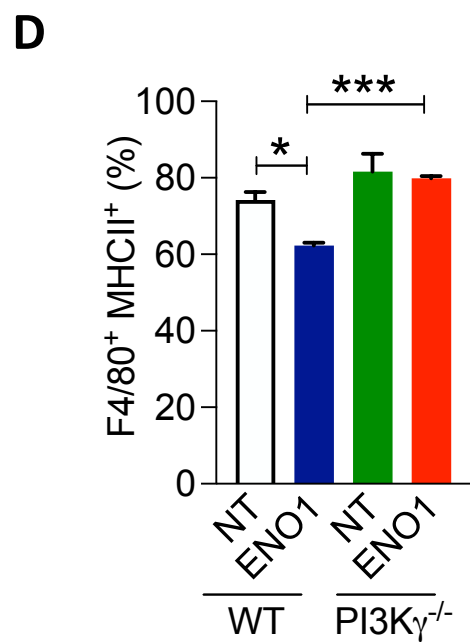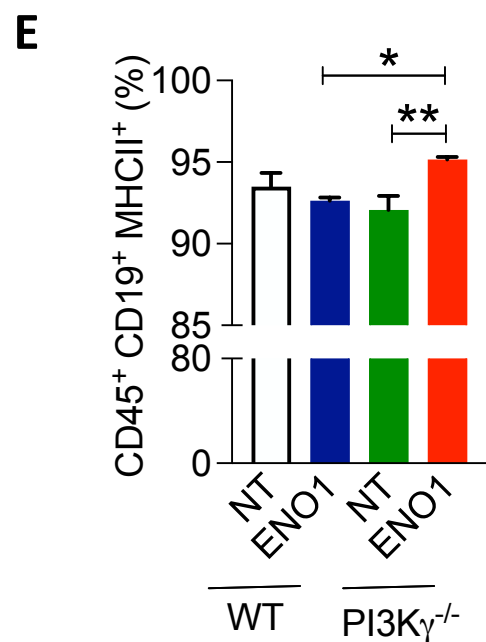

Supplementary Figure 4

Supplement: Supplementary file 4 — Additional file 4. Flow cytometry analysis of activated dendritic cells (A) and macrophages (B) and activated macrophages expressing MHC II (C) in splenocytes from C57/Bl6 WT and PI3Kg-/- vaccinated or unvaccinated mice. Flow cytometry analysis of MHC II expression of macrophages (D) and B cells (E) in lymph nodes from C57/Bl6 WT and PI3Kg-/- vaccinated or unvaccinated mice. Treatment groups: white bars, PBS-DMSO; green bars, TG100-115 inhibitor; blue bars, ENO1 DNA vaccine; red bars, ENO1+TG100-115 (combined treatment). In all experiments, the number of mice per group was between 3 and 4; graphs report the mean±SEM values, and statistical significance using one way ANOVA test is shown, * p < 0.05, ** p <0.001, and *** p < 0.0001 [file 13046_2024_3080_MOESM4_ESM.pdf]
