# Supplementary material for: PI3Kγ inhibition combined with DNA vaccination unleashes a B-cell-dependent antitumor immunity that hampers pancreatic cancer
Source: J Exp Clin Cancer Res. 2024 Jun 1;43:157. doi: 10.1186/s13046-024-03080-1 (PMC11143614; doi:10.1186/s13046-024-03080-1)
Supplement: Supplementary file 5 — Additional file 5. [file 13046_2024_3080_MOESM5_ESM.docx]

**Supplementary File**

**Tumor lesion evaluation and immunohistochemical staining.**

The pancreases of mice were formalin-fixed and paraffin-embedded, and 4 µm-thick slices were stained with hematoxylin and eosin. Tissues were scanned and the mean diameter and number of all neoplastic lesions from PanIN to invasive PDA were analyzed using NDP view2 (Hamamatsu, Shizuoka, Japan). For immunohistochemistry staining, peroxidase activity was inhibited by exposure to 3% hydrogen peroxide aqueous solution for 10 min. Samples were pre-treated by microwave antigen retrieval using EDTA buffer, citrate pH 6 (Agilent Dako, Milan, Italy) or protease (Ventana, Segrate, Italy), and incubated with the following antibodies: CD4 (Abcam, Cambridge, UK, diluted at 1:1000), CD8 (Affimetrix eBioscience, Milan, Italy, diluted at 1:3000), CD31 (Abcam, diluted at 1:2000), NG2 (Abcam, diluted at 1:200), B220 (Invitrogen, Milan, Italy, diluted at 1:2000), Ki-67 (Abcam, diluted at 1:200) or F4/80 (AbD Serotec, Milan, Italy, diluted at 1:100), CD80 (Abcam, diluted at 1:500) and CD163 (Abcam, diluted at 1:1000) for 30 to 48 min at room temperature. The rabbit EnVision system (Agilent Dako), anti-mouse-horseradish peroxidase (HRP) (GE Healthcare) or biotinylated goat anti-rat (Bio-Rad, Hercules, CA, USA) followed by HRP-conjugated streptavidin (Millipore, Milan, Italy), were used prior to diaminobenzidine tetrahydrochloride (Agilent Dako) incubation. Negative and positive controls were performed to set up the staining protocol. All slides were stained for the same antigen, together with the same antigen-retrieval buffer and antibody dilution. Tissues were examined in a double-blind fashion and digital images of representative areas were taken. Positive cells in the tumor area of the pancreatic tissues sections were counted and classified as absent (score 0.2, 0 positive cells), scarce (score 0.4, 1-10 positive cells), moderate (score 0.6, 10-50 positive cells), strong (score 0.8, 50-100 positive cells), or huge (score 1, >100 positive cells) on sections stained with the different antibodies. In addition, for the antigens showing statistical differences between treatments, the number of positive cells per square millimeter in tumor area were analyzed using the QuPath software (Version 0.5.0).

**mRNA extraction from formalin-fixed paraffin-embedded tissues (FFPE) and qPCR.**

Sections of whole tumors were cut and processed using the RNeasy FFPE kit (Quiagen) as indicated by the manufacturer’s instruction. To produce cDNA from 1 μg of mRNA, the iScript Reverse-Transcription Kit (Bio-Rad) was used, following the manufacturer’s instructions. qPCR was performed using SYBR Green Mix (Applied Biosystems by Thermo Fisher) using the following primers:

Col1a1, 5’-CCCCTGGTCAAGATGGTC-3; 5’-CTCCAGCCTTTCCAGGTTCT-3;

aSma, 5’-GTTCAGTGGTGCCTCTGTCA -3’; 5’-ACTGGGACGACATGGAAAAG -3’;

Fap, 5’-CGCGTAACACAGGATTCACT-3’; 5’-TCGGAGGAGAGTTTCCAATG -3’;

Postn, 5-AGCAAGCAGGGAAGGAATG-3; 5- GAGGCTGAGGAAGATGCAAAG-3;

Cd8, 5-GGATTGGACTTCGCCTGTGA-3; 5-CTTTCGGCTCCTGTGGTAGC-3;

Nfkb, 5-TCTGGCACAGAAGTTGGGTC-3; 5-GGTACCCCCAGAGACCTCAT-3;

Gzmb, 5-GACAACACTCTTGACGCTGG-3; 5-TGATCTCCCCTGCCTTTGTCC-3;

Cxcl10, 5’-ATGACGGGCCAGTGAGAATG-3’; 5’-TCGTGGCAATGATCTCAACAC-3’;

Gmcsf, 5’-CCACCTATGCGGATTTCATAGA-3’; 5’-TCCAAGTTCCTGGCTCATTAC-3’;

Mcsf, 5’-GCTCTAGCCGAGATGTGGTG-3’; 5’-AATCATCCCAAGCCAAGCCA-3’;

Gcsf, 5’-CTGTTTGCTCCTAGCCCTGC-3’; 5’-TGTAGGTGGCACACAACTGC-3’;

B2m, 5’-CTCGGTGACCCTGGTCTTTC -3’; 5’-TTGAGGGGTTTTCTGGATAGCA-3’;

Nos2, 5’-CTTTGCCACGGACGAGAC -3’; 5’- TCATTGTACTCTGAGGGCTGAC-3’;

Ifng, 5’-ATCTGGAGGAACTGGCAAAA -3’; 5’- TTCAAGACTTCAAAGAGTCTGAGGTA-3’;

Cd86, 5’-GAAGCCGAATCAGCCTAGC -3’; 5’- CAGCGTTACTATCCCGCTCT-3’;

Il12b, 5’-AAGGAACAGTGGGTGTCCAG-3’; 5’- GTTAGCTTCTGAGGACACATCTTG-3’;

Arg1, 5’-GAATCTGCATGGGCAACC-3’; 5’- GAATCCTGGTACATCTGGGAAC-3’;

Ym1, 5’-AAGAACACTGAGCTAAAAACTCTCCT-3’; 5’- GAGACCATGGCACTGAACG-3’;

Pd1, 5’-CCCACTTCAAGTTCAACTGTCT-3’; 5’-TTCTGAAGCAAAGAATGAAAGTG-3’;

Cd14, 5’-AGCAACAAGCCAAGCACAC-3’; 5’-AAAGAAACTGAAGCCTTTCT-3’;

Il10, 5’-CAGAGCCACATGCTCCTAGA-3’; 5’-TGTCCAGCTGGTCCTTTGTT-3’;

Gapdh, 5’-CATCACTGCCACCCAGAAGACTG -3’; 5’ ATGCCAGTGAGCTTCCCGTTCAG-3’.

Heat-map represents the fold change of 2^-deltaCt values of the treated groups compared to the untreated group. *Gapdh* was used as a housekeeping gene to obtain delta-Ct values used for the analysis.

**Serological Proteome Analysis (SERPA).**

Sub-confluent K8484 cells were washed twice in PBS, pelleted and solubilized in a solution containing 9 M urea, 4% CHAPS, 1 mM Na_3_VO_4_, 80 mM DTT, protease inhibitors and nuclease. Samples were centrifuged and clear supernatants were removed, quantified with detergent compatible protein assay kit (Bio-Rad, Hercules, CA, USA) and stored at -80°C until required for analysis.

Proteins (200 μg per sample) were loaded on ready-made IPG strips (7 cm IPG strips, pH 3-10NL) (Bio-Rad). The second dimension was run on 4-12% polyacrylamide gels (ThermoFisher, Waltham, MA, USA). Proteins from 2DE gels were transferred to nitrocellulose membranes (GE Healthcare, Milan, Italy). Membranes were blocked with 5% w/v non-fat dry milk (Microgem, Pozzuoli, NA, Italy) in PBST (PBS supplemented with polyoxyethylene sorbitan monolaurate [Tween-20] 0.1% v/v) for 1 h, and then probed with a 1:100 dilution of mouse serum overnight at 4°C. Membranes were washed with PBST, incubated for 1 h with anti-mouse IgG1 or anti-mouse IgG2b HRP (ThermoFisher) diluted at 1:5000 and 1:2000, respectively, or with anti-mouse IgG2c HRP (Jackson ImmunoResearch, West Grove, PA, USA) diluted at 1:5000. Immunoreactivity was detected with an enhanced chemiluminescence kit (Pierce, Rockford, IL, USA). Images were acquired using ChemiDoc (Bio-Rad).

**Magnetic resonance imaging (MRI).**

Before MRI analysis, mice were anesthetized with Zoletil and Xylazina. Mice were then placed prone in a solenoid Tx/Rx coil with an inner diameter of 35 mm. After the scout image acquisition, an axial T_2_‐weighted (T_2w_) anatomical image was acquired with a Fast Spin Echo sequence (TR = 2500 sec; TE = 44 msec; number of slices = 10; slice thickness = 1.5 mm; FOV = 40 mm; matrix = 152 × 160; four averages; acquisition time = 3 min 20 sec). DWI‐MRI was performed as previously described (10) using a Spin‐Echo sequence with seven *b*‐values (0, 50, 100, 150, 200, 400, 600 s/mm^2^) with the following parameters: TR = 500 ms; TE = 15.4 ms; number of slices = 10; slice thickness = 1.5 mm; FOV = 40 mm; matrix = 128 × 128; 1 = average; acquisition time = 7 m 28 sec. Diffusion-weighted images (DWI) were used to provide the Apparent Diffusion Coefficient (ADC) maps and the IVIM-based (intravoxel-incoherent motion) parameters, including true diffusion coefficient (D) maps, by fitting DWI‐MRI signal intensity as a function of *b*‐values in MATLAB, working on a pixel-by-pixel basis (11). ADC, and D values were calculated by superimposing parametric maps on the selected regions of interest corresponding to the pancreas drawn in T_2w_ anatomical images.

**Flow cytometry and western blot analysis of lymph nodes from treated mice.**

Lymph nodes (2x10^5^ cells) from untreated or ENO1-vaccinated C57/Bl6 or PI3Kγ^-/-^ mice were dissociated, washed with PBS/0.2% BSA/0.01% NaN3, stained with CD4 (Miltenyi, Germany) BCL6, CXCR5 and CD40L monoclonal antibodies (all Biolegend, San Diego, California, USA).

For western blot analysis, cells isolated from inguinal and popliteal lymph nodes of WT and PI3Kγ^-/-^ mice ENO1-vaccinated or untreated as previously described, were pelleted and solubilized in GST buffer supplemented with 1 mM Na_3_VO_4_, 1 mM PMSF, 10 mM NaF, protease and phosphatase inhibitors and nuclease. Clear supernatants were removed, quantified with detergent compatible protein assay kit (Bio-Rad) and stored at -80°C until analysis. Total lysates (30 μg per sample) were separated using 4-12% polyacrylamide gels (ThermoFisher). Separated proteins were transferred to nitrocellulose membranes (GE Healthcare). Membranes were then blocked with 5% w/v BSA (Bovine Serum Albumin, Sigma-Aldrich, Saint Louis, MO, USA) in PBST for 1 h. This was followed by incubation with a 1:500 dilution of anti-BCL-6 antibody (Abcam, Cambridge, UK) or with a 1:1000 dilution of anti-Actin antibody (Sigma-Aldrich, Saint Louis, MO, USA) both in PBST with 3% w/v BSA, overnight at 4°C. Membranes were then washed with PBST and incubated for 1 h with anti-rabbit IgG HRP (Cytiva, Marlborough, MA, USA) diluted at 1:10000. Immunoreactivity was detected with an enhanced chemiluminescence kit (Pierce). Images were acquired using ChemiDoc (Bio-Rad). The intensity of the bands was evaluated with Fiji (Fiji Is Just ImageJ) open-source image processing package, and BCL6 expression levels were calculated by normalizing the intensity of the bands against housekeeping protein levels.

**Enzyme-Linked Immunosorbent Spot Assay (ELISpot)**.

Plates were developed using 3-amino-9-ethylcarbazole substrate according to the manufacturer’s instructions, and spots were quantified with the microplate reader, along with a computer-assisted image analysis system (Immunospot; CTL Europe, Bonn, Germany). The number of spots was calculated by subtracting the number of spots in medium only (background) from that in the presence of stimuli.

**Enzyme-Linked Immunosorbent Assay (ELISA).**

Recombinant human ENO1 (rENO1, Sigma-Aldrich, Milan, Italy) protein was coated at 2 μg/ml in 0.1 mol/L Na_2_CO_3_ onto flat-bottomed plates and incubated overnight at 4°C. Mouse serum samples were diluted at 1:50 in PBS followed by incubation with anti-IgG HRP diluted at 1:8000 for 1 h and tetramethylbenzidine (Tebu Bio, Magenta, Italy) for 25 min. The 72/1.11 monoclonal antibody was used as a positive control (kindly provided by P. Migliorini, University of Pisa, Pisa, Italy). DELTA values were calculated by subtracting the optical density of coated wells from the uncoated wells to account for background signal.

**Antibody-dependent cell cytotoxicity (ADCC).**

A total of 1×10^4^ K8484 target cells were stained with 2 µM of carboxyfluorescein diacetate succinimidyl ester (CFSE; Molecular Probes) following the manufacturer’s instructions. Labeled cells were incubated overnight at 37°C with splenocytes from untreated C57BL/6 mice as effector cells (200:1, 100:1 and 50:1 as the effector:target [E:T] ratios) and sera from ENO1-vaccinated mice (at a 1:50 dilution). Cells were then harvested, stained with 1 μg/ml 7-Amino-Actinomycin D (7-AAD, BD Bioscience), acquired on a BD FACSVerse and analyzed using BD FACSuite™ software (Becton Dickinson). Percentage killing was obtained by measuring the % of 7-AAD^+^ dead cells among the CFSE^+^ targets, and % ADCC for each serum sample was calculated with the formula [(dead targets in sample (%) – spontaneously dead targets (%))/(dead target maximum-spontaneously dead targets (%))] × 100. Spontaneous release was obtained by incubating target cells in medium with serum but without splenocytes, while maximum release was obtained after treating target cells with 1% saponin (12).

**Serum-binding potential (SBP).**

Sera from untreated and empty or ENO1-vaccinated mice were used to stain K8484 cells, which were analyzed by flow cytometry after dilution by 1:50. Briefly, 1x10^5^ cells were washed with PBS/0.2% BSA/0.01% NaN3 and incubated with diluted sera for 1 h at 4°C. After two washes, cells were incubated with an APC-conjugated anti-mouse antibody (1:200; Biolegend; Prodotti Gianni, Milan, Italy) on ice for 30 min. Following washing, 1x10^4^ cells were analyzed with an Accuri C6 using FlowJo software (both BD Biosciences, Buccinasco, MI, Italy). The antibody titer is expressed as serum binding potential x 10^-3^/mL, calculated as previously described in detail (13).
